# Blood Group Discrepancies at a Regional Blood Center

**Published:** 2020-01-01

**Authors:** Hayedeh Javadzadeh Shahshahani, Azam Hayati

**Affiliations:** 1 Blood Transfusion Research Center, High Institute for Research and Education in Transfusion Medicine, Tehran, Iran; 2 Blood Transfusion Research Center, High Institute for Research and Education in Transfusion Medicine, Yazd, Iran

**Keywords:** Blood group discrepancy, Blood donors, ABO blood typing

## Abstract

**Background:** Blood group testing is an important part of supplying safe blood components in blood transfusion centers. Blood group discrepancy develops when reactions in forward grouping do not correspond with reverse grouping or if the preceding and recent results do not match. This study aimed to evaluate ABO blood group discrepancies among blood donors of Yazd, Iran.

**Materials and Methods: **In this cross-sectional study, data of blood donors were obtained from the integrated database of Yazd Blood Transfusion Center during a period of eight years (2010 – 2017). Tube testing was used for determining the ABO blood groups. A serological workup was performed for diagnosis and determination of the discrepancy. Confirmation of the results was accomplished by the reference laboratory of immunohematology.

**Results: **Blood group discrepancies were detected in 130 (0.04%) out of 322,222 donations. Technical/Clerical errors leading to ABO discrepancy were noticed in 12 (9.3%) cases. The most frequent cause of ABO discrepancies in forward grouping was subgroups of A Antigen (44.6%) and in reverse grouping was cold autoantibody (23.9%). There were 11 (8.4%) cases with alloantibodies. Two blood donors with rare Bombay phenotype and p blood group were also identified.

**Conclusion** **: **For minimizing Technical/Clerical errors, accurate blood donor or sample identification programs should be implemented. All cases of blood group discrepancies should be carefully investigated, and blood donors should be informed appropriately.

## Introduction

 Providing safe blood components needs many different laboratory tests including ABO blood grouping. Donor blood samples are routinely typed for ABO at the time of donation. ABO typing requires both antigen typing of red cells for A and B antigen (red cell or forward typing) and screening of plasma for the presence of anti-A and anti-B isoagglutinins (plasma or reverse typing). Both red cell and plasma typing are required for donors because each grouping performs a confirmatory test for the other ^1^. It is critical for recipient safety that ABO typing should be performed, recorded, and interpreted precisely. The risk of acute hemolytic transfusion reaction due to transfusion of ABO-incompatible blood components is at least 100 times more than the risk of transfusion-transmitted infections and may lead to serious complications in the recipient^2^. Blood group discrepancy develops when the results of red cell typing do not match with plasma typing, or if the previous and present results do not match^3^. ABO discrepancies may be due to clerical errors or technical problems with a sample or during testing. Intrinsic problems within red cells or plasma may also lead to ABO discrepancies. 

Although many advances have been presented for ABO blood grouping, discrepancies still occur. Since the ABO system is the most important blood group system in relation to transfusions, misinterpreting ABO discrepancies could be life-threatening to patients. Therefore, the interpretation of the ABO blood group must be delayed and the blood unit must be quarantined and cannot be released for transfusion until the discrepancy has been resolved^1^. 

The frequency of ABO discrepancies and their causes vary in different studies ^3^^-^^5^. The aim of the present study was to determine the frequency and causes of ABO blood grouping discrepancies among blood donors in a regional blood center in Yazd, Iran.

## MATERIALS AND METHODS

 This cross-sectional study was conducted in the immunohematology laboratory of Yazd Blood Transfusion Center from March 2010 to March 2017. Demographic data of donors and previous history of blood donation were obtained from integrated software of Yazd Blood Transfusion Center. All blood donor samples received during the study period were analyzed. The exclusion criteria were deferred donors. All donor samples were tested for ABO typing using the tube method. Monoclonal antisera: anti-A, anti-B (Iranian Blood Research and Fractionation, Tehran, Iran) and in-house cells (group A1, B, and O reagent red blood cells) were used for forward and reverse grouping, respectively. The tests were performed according to standard operational procedures (SOPs) of the Iranian Blood Transfusion Organization (IBTO). 

In all discrepant cases, technical/clerical errors were investigated first. Repeat ABO typing was performed on the same sample and on a new sample using the standard tube method. After ruling out technical/clerical errors, problems with RBCs or plasma were studied. Monoclonal antisera anti-A, anti-B, anti-AB (CE-IMMUNDIGNOSTIKA GmbH, Eschelbronn, Germany) and in-house donor red blood cells (A1 cell, B cell, and O cell) were used for forward and reverse grouping. Supplementary reagents used included anti-A1 lectin and anti-H lectin (CE-IMMUNDIGNOSTIKA GmbH, Eschelbronn, Germany) along with in-house pooled A2 cells wherever required. Monoclonal antisera (anti-A, anti-B, and anti-AB) and in-house pooled cells used for testing by the tube method underwent daily quality control according to the SOPs of IBTO before use. Three-cell antigen panel (IBTO mini-panel) was used for the antibody screening procedure. An IBTO-homemade 11-cell antibody panel and selected cells were used for antibody identification by standard tube method ^[^^6^^]^. The American Association of Blood Banks (AABB) Technical Manual was used for resolving ABO discrepancies ^1^. Cases with true discrepancy were also referred to IBTO reference laboratory of immunohematology for confirmatory tests. Data were analyzed using SPSS 17.5 software (IBM Corporation, New York, NY, USA). The study was approved by the Ethics Committee of Shahid Sadoughi University of Medical Sciences.

## Results

Table 1 shows ABO blood group distribution and demographic details of blood donations with or without blood group discrepancy during the study period. Blood group discrepancies were discovered in 130 cases (0.04%). Blood group discrepancies were found more frequently in A and AB blood group donations.

**Table 1:** ABO blood group distribution and demographic details of blood donations with or without blood group discrepancy

**Year**	**No. of ** **donations**	**Sex** Male %	**Age** **Averag** **e**	**Blood group (%)**				**No. of ** **donations with ** **blood group ** **discrepancy**	**Sex** **Male** **%**	**Age** **Average**	**Blood group (%)**			
				**A**	**B**	**AB**	**O**				**A**	**B**	**AB**	**O**
2010	38908	96	42	26	31	9	34	17	94	38	47	23	18	12
2011	40262	97	42	25	31	9	35	17	100	36	47	12	30	11
2012	43129	97	41	25	31	9	35	7	100	40	43	14	29	14
2013	38819	97	41	25	32	9	34	14	100	37	43	15	28	14
2014	39664	97	40	26	31	9	34	17	88	31	35	18	35	12
2015	40766	97	39	26	31	9	34	24	95	36	50	9	33	8
2016	39973	97	39	26	30	9	35	17	94	38	47	23	24	6
2017	40701	97	38	26	30	9	35	17	82	34	47	23	18	12
Total	322222							130						

Nine percent of ABO blood group discrepancy occurred due to technical/clerical errors. Donor misidentification occurred when the blood donor presented another donor ID card. It also occurred when donor data was recorded in another donor’s datasheet erroneously, which could happen when another record in the registration system had a similar name or date of birth. 

Mislabeling of sample tubes was another reason for technical errors. Approximately 47% of discrepant results were noted with the forward blood grouping followed by the reverse blood grouping (44%). Subgroups of A Antigen (44.6%) and cold autoantibodies (23.9%) were the most frequent reasons for blood group discrepancy. Table 2 shows the distribution of reasons for ABO blood group discrepancy.

**Table 2:** Distribution of reasons for ABO blood group discrepancy

** Type of discrepancy**	**Causes of ABO discrepancy**	**N (%)**	**Total** **N (%)**
Technical/clerical errors	Blood donor identification error	5(3.9)	12(9.3)
	Mislabeling of sample tubes	7(5.4)	
Forward blood grouping	Subgroup of A Antigen (n); A_2 _(31), A_3_ (7), A_2_B (20)	58(44.6)	61(46.9)
	Subgroup of B Antigen (n); B_3_ (3)	3 (2.3)	
Reverse blood grouping	Cold-reacting autoantibodies	31(23.9)	57(43.8)
	Weak/Low avidity anti-B antibodies	12(9.2)	
	Alloantibodies(n); anti-M (5), anti-P1 (4), anti- PP_1_P^k^ (1), anti-H (1)	11(8.4)	
	Weak/Low avidity anti-A, B antibodies	3(2.3)	
Total			

Table 3 shows the serological reactions of the subgroups of ABO blood groups. One case of Bombay phenotype was found during the study period. This rare blood group was discovered in reverse blood grouping (Table 3). Additionally, we detected one case of rare p phenotype with the presence of naturally occurring antibody against missing antigens (anti-PP_1_P^k^) (Table 2). 

**Table 3:** The strength of serological reactions of the ABO blood subgroups

**Red cell ** **Phenotype**	**Forward blood Grouping** **X±SD (range)**					**Reverse blood Grouping** **X±SD (range)**		
	**Anti-A**	**Anti-B**	**Anti-AB**	**Anti-A1**	**Anti-H**	**A1 cells**	**B cells**	**O cells**
A_2_	2.13±0.57 (2+/4+)	0	3.20±0.41 (3+/4+)	0	2.20±0.41 (2+/3+)	0.1±0.4 (0/2+)	4+	0
A_3_	1.43±0.53 (1+/2+)^ mf^	0	1.43±0.53 (1+/2+)^ mf^	0	3+	0	4+	0
A_2_B	2.80±0.89 (1+/4+)	4+	4+	0	1+	0.4±0.76 (0/2+)	0	0
B_3_	0	1+^ mf^	2+^ mf^	NA	4+	4+	0	0
Bombay (O_h_)	0	0	0	0	0	4+	4+	4+

1+ to 4+ = agglutination of increasing strength; mf = mixed-field agglutination; 0 = no agglutination; NA = Not Applicable

## Discussion

 Accurate blood donor identification and collection of a properly labeled blood specimen from the intended donor is critical for safe blood transfusion. ID card with a photograph is currently used for registration of first-time donors in Yazd blood transfusion center. For repeat donors, the clerical staff should ask the donor to spell his or her name and to state his or her date of birth or ID number to compare and verify the donor ID history in the computer system with the verbal information provided by the donor. If the information does not match, the discrepancy must be resolved before registration. However, in five cases (4%), blood donor identification error occurred. Using biometric identification such as fingerprints, retinal scans, or iris scans for verifying first-time and repeat donor identity can prevent identification errors. Mislabeling of sample tubes occurred in seven cases (5.4%). The phlebotomist is required to identify the donor by carefully examining the information on the donor form before collecting a specimen. The phlebotomist must identify the potential donor and must ensure that the correct donor information is placed on the label of the sample that is collected. In the study of Kaur et al., technical errors were found in five (18%) cases^4^. "Positive" donor ID systems may accomplish the identification of the donor, specimens collected, blood unit identification, and linkage of blood components to their intended donor(s).

The frequency of blood group discrepancy in different studies ranged from 0.02 to 0.06% ^3^^-^^5^^,^^7^. that was similar to the results of the present study (0.04%). Subgroups of A antigen (44.6%) were the most frequent reasons for blood group discrepancy in our study. Similar results have been reported by Kaur et al ^4^. However, Makroo et al. have reported that subgroups of A antigen (29%) were the second most common cause in their study ^5^. In Sharma et al. study, subgroups of A Antigen accounted for 19.6% of discrepancies^3^ Subgroups of A antigen account for a small proportion of the A population. These subgroups have fewer antigen sites on the surface of the red blood cell. As a result, they show weakened (or missing) reactions when tested with commercial antisera. Extending incubation time may enhance the reaction. Testing with a monoclonal blend of anti-A,B - anti-A1, anti-H lectin; Testing A, B, and H substances in the saliva of secretors, Adsorption Elution test, Family Study, and DNA-based study are other tests used for determining the blood group. Two out of 31 (6.5%) subgroup A2 and 5 out of 20 (25%) subgroup A2B donors developed anti-A1 antibody in their serum. The antibody is a naturally occurring IgM. For resolving anti-A1 discrepancy, typing patient RBCs with Anti-A1 lectin, repeating reverse grouping with 3 A1 Cells and repeating reverse grouping with 3 A2 Cells could help. 

Three cases (2.3%) of B subgroups were detected in the present study. Subgroups of B antigen are less than A subgroups. In Sharma et al. study, B subgroups were reported to be 4%^3^. Other studies have reported that B subgroups were more common in India in comparison to some other populations ^7^^,^^8^. 

Cold autoantibody (23.9%) was the second most frequent reason for blood group discrepancy in the present study. In Makroo et al study, cold-reacting autoantibody (57%) was the most common cause of ABO discrepancy. In most people, cold-reacting autoantibody will not react above 10°-15°C. However, sometimes a donor will develop cold-reacting auto-antibodies that react at room temperature and appear as “extra” antibodies on reverse typing. These harmless autoantibodies are IgM and react best at 4°C. For the resolution of the discrepancy, screening for antibodies using Screening Cells and an autocontrol is needed. The autocontrol will be positive. Special procedures such as prewarming techniques or autoadsorption could also be used.

We observed that 11 blood donors had cold-reacting alloantibodies. Similar results have been reported in Makroo et al or Sharma et al studies^3^^,^^5^. The prevalence of anti-M was reported to be 1 in 2500 to 5000 in blood donors ^9^. Anti-M or anti-P1 antibodies were found in nine donors in our study. These are naturally occurring IgM antibodies. Most of anti-M or anti-P1 antibodies show optimal reactivity at room temperature or below and are not active at 37-degree centigrade and are considered to be clinically insignificant ^10^. However, anti-M in one case of the present study reacted at 37-degree centigrade, therefore, considered to be clinically significant because of the potential to cause hemolytic transfusion reaction. 

Two donors had rare alloantibodies (anti- PP_1_P^k^, anti-H). Anti-PP_1_P^k^ (historically known as anti-Tj^a^) is a potent hemolysin in the rare p phenotype and is associated with hemolytic transfusion reactions^11^. Anti-H is a strong reacting alloantibody in the case of Bombay phenotype. O_h_ individuals lack all ABH antigens; therefore, they possess natural antibodies to A, B, and H. In forward ABO typing, these individuals initially are typed as group O. In reverse typing, anti-H alloantibody present in O_h_ individuals react strongly with group O red cells, which are rich in H antigen (Table 3). Because the anti-H in Bombay phenotype is capable of activating complement and causing red cell hemolysis, it is clinically significant and associated with acute hemolytic transfusion reactions. In the present study, the O_h_ phenotype was confirmed by demonstrating an absence of H antigen on red cells using anti-H Lectin.

Alloantibodies in Bombay and p phenotypes reacted strongly with all panel cells in room temperature, 37-degree centigrade, and antihuman agglutination phase. The strength and test phase of reactions were uniform for all of the red cells tested. However, the autocontrol was nonreactive. Therefore, an alloantibody to a high-prevalence antigen was considered. By testing selected red cells of rare phenotypes and by typing the patient's autologous red cells with antisera to high-prevalence antigens, antibodies to high-prevalence antigens were identified.

These rare alloantibodies are highly clinically significant and are capable of fixing complement and causing hemolytic transfusion reactions. As a result, rare patients with alloanti-H or anti- PP_1_P^k^ must be transfused with antigen-negative, crossmatch-compatible RBCs^12^. Because of difficulties in finding compatible units in the local population, individuals with these rare phenotypes should be encouraged to donate their blood to be stored frozen for future possible needs^13^. Detection of these rare phenotypes helps for improving the national rare blood donors program.

Weak/Low avidity (anti-B or anti-A, B) antibodies were found in fifteen donors and accounted for 11.5% of discrepancies. In Makroo et al study, weak antibody caused 7% of discrepancies, while in Sharma et al study, weak antibody was the most common cause of ABO discrepancy (58.8%)^3^^,^^5^. For resolving weak or missing antibodies, incubation of serum testing for 15 minutes at room temperature to enhance antibody reactions and testing serum at 4°C for 5 minutes along with autologous control should enhance the reactivity of the antibodies.

In Iranian blood transfusion centers, a donor unit with blood group discrepancy should not be labeled or released for transfusion and must be discarded. However, there is no integrated plan for informing these donors about their blood group. For reporting their accurate blood group, special methods such as family studies or molecular techniques may also be required. Such blood donors should be notified and should also be issued a specific blood group card that clearly states their respective donor or recipient status^7^. 

## CONCLUSION

 In general, for decreasing the chance of developing hemolytic transfusion reactions due to clerical errors, accurate methods for blood donor or sample identification should be implemented; all cases of blood group discrepancies should be carefully investigated, and blood donors with discrepancies should be informed appropriately about their blood group as a blood donor or blood recipient.
